# Exploring the electronic structure, mechanical behaviour, thermal and high-temperature thermoelectric response of CoZrSi and CoZrGe Heusler alloys

**DOI:** 10.1038/s41598-023-48316-w

**Published:** 2023-12-21

**Authors:** Bharti Gurunani, Dinesh C. Gupta

**Affiliations:** Condensed Matter Theory Group, School of Studies in Physics, Jiwaji University, Gwalior, 474011 India

**Keywords:** Condensed-matter physics, Theory and computation

## Abstract

By using density functional theory, we have explored the structural, electro-mechanical, thermophysical and thermoelectric properties of CoZrSi and CoZrGe Heusler alloys. The ground state stability was determined by optimising the energy in various configurations like type I, II, and III. It was found that these alloys stabilized in the ferromagnetic phase in type I. We employed the Generalised Gradient Approximation and modified Becke-Johnson potentials to explore the electronic structure. The band structures of each of these Heusler alloys exhibit a half-metallic nature. Additionally, the computed second-order elastic parameters reveal their ductile nature of them. To understand the stability of the alloys at different pressures and temperatures, we investigated various thermodynamic parameters using the Quasi-Harmonic Debye model. We obtained the transport coefficients using the Boltzmann theory. Our findings indicate that these alloys can be used in spintronics and thermoelectric domains.

## Introduction

Due to the growing global population and the adoption of modern lifestyles, energy consumption has increased, resulting in a demand for safe and eco-friendly energy resources across all regions and scales. The global energy crisis has prompted researchers to explore some novel and appropriate methods for enhancing energy conversion methods. However, traditional energy sources, although efficient, are not considered reliable future energy resources due to pollution and the risk of radiation accidents. Thermoelectricity, which involves converting heat directly into electricity, is a potential solution, and the materials that enable this technology are referred to as thermoelectric materials^1,2^. Thermoelectric materials can produce thermo e.m.f when there is a temperature gradient, which makes it a suitable option for converting waste heat energy into electric energy. Thermoelectric Modules (TEM) are used to eliminate the need for toxic materials, complicated designs, and fuel, which has led to this technology being recognized as the environmentally friendly Green Technology for energy production^3^. TEMs offer an alternative significant advantage over thermal power plants in that they do not require to produce heat and can be easily installed in any region where waste heat is available. As a result, they have gained popularity in various sectors, including domestic, commercial, and automobile technology, where waste heat centres can be used to generate electric energy^4–6^. The figure of merit (ZT) of thermoelectric materials is highly valued, and there is no limit. However, only a few alloys such as Bi2Te3 and CoSb3 have ZT = 1 in recent years, making them the most effective thermoelectric materials^7–9^. These accomplishments have led to the introduction of a novel group of materials, known as Heusler alloys. Friedrich Heusler first introduced these compounds in 1903, and they are now recognized as emerging materials in many current research fields. Heusler alloys have a promising range of applications that extend from semiconductors to superconductors. Their outstanding properties include the capacity to regulate the concentration of valence electrons via partial element exchange. In the presence of transition elements (X and Y) and the p-block elements (Z), these compounds have cubic structures (FCC) and can form half Heuslers (XYZ) and full Heuslers (X_2_YZ). It is also possible to create a new type of Heusler compound, called Quaternary Heusler (QH) compounds, by occupying any appropriate space between the Half-Heusler or Full Heusler alloys. Several half and full Heuslers are identified as effective materials for superconductivity, ferromagnetic, spintronics, and topological devices^10–18^. It has been noted that effective thermoelectric materials exhibit characteristics of both phonon crystal and electron crystal behaviour^19^. To achieve good thermoelectric performance, the alloy must have thermal properties similar to that of an amorphous glass and electrical attributes similar to that of a crystal. Heusler Compounds have all of the required properties in a narrow bandgap semiconductor, including, mechanical strength, semiconducting behaviour, and good thermal and electrical properties, while reasonably priced^20–24^. The thermoelectric properties of Heusler alloys make them suitable for high-temperature applications. Various effective Heusler alloys, such as Fe_2_VAl, Fe_2_TiSi, FeVSb, FeNbSb, TiCoSb and TiNiSn, are less exclusive than traditional efficient thermoelectric materials^25^. Recently, a few Heusler compounds, such as RuTaSb, Hf1-xTixCoSb0.8Sn0.2, etc., have been studied and found to exhibit better electronic properties and thermoelectric performance than predictable PbTe and Bi2Te3 based thermoelectric materials^26–29^. In 2013, the Half Heusler compounds MNiSn and MCoSb (M = Ti, Zr, Hf) were explored by Shuo Chen et al., resulting in a Figure of Merit (ZT) greater than 1 at 700 K^30,31^. According to recent reports, a few n-type and p-type half Heuslers with the values of ZT in the 900–1000 K range have ZT values of 1.05 and 0.8, respectively^32^. The research conducted by Evren G Özdemir and Ziya Merdan contributes significantly to the understanding of the CoZrGe half-Heusler compound's electronic properties. The findings of this study can potentially impact the development of future spintronics applications by providing valuable data on the compound's half-metallic behavior^33^.

## Calculation method

We used the Wien2k code and Density Functional Theory (DFT) method to explore the theoretical aspects of CoZrSi and CoZrGe alloys^34^. To explore the exchange–correlation corrections, the Generalized Gradient Approximation (GGA) in Perdew–Burke–Ernzerhof (PBE) was used along with modified Becke-Johnson potentials (mBJ)^35^. Our research revealed that the measured value of the lattice constant was nearly similar to that of the optimized lattice constant at the minimum energy. For energy convergence, the cut-off value *R*_*MT*_*K*_*max*_ was selected as 7.0, where *R*_*MT*_ is the smallest muffin-tin sphere radius and *K*_*max*_ is the largest reciprocal lattice vector. An electron density difference is less than 0.0001e per f.u., where ‘e’ is the electron charge, is considered to be converged in the self-consistent calculation. Employing 3000 K-points in the first Brillouin zone (BZ), each atomic sphere was harmonically enlarged to an *I*_*max*_ = 10. For the thermodynamic calculations, we used the Quasi-Harmonic Debye model^36^. The transport properties are estimated within the semi-classical Boltzmann theory framework using the BoltzTraP code and a constant time relaxation approximation^37^. The elastic parameters were calculated to examine the material's mechanical stability by using the elastic code^38^.

## Result and discussion

The structural, electro-mechanical, thermophysical and thermoelectric properties of Cobalt-based half Heusler alloys CoZrSi and CoZrGe are elaborated below.

### Structural properties

**Figure 1:**
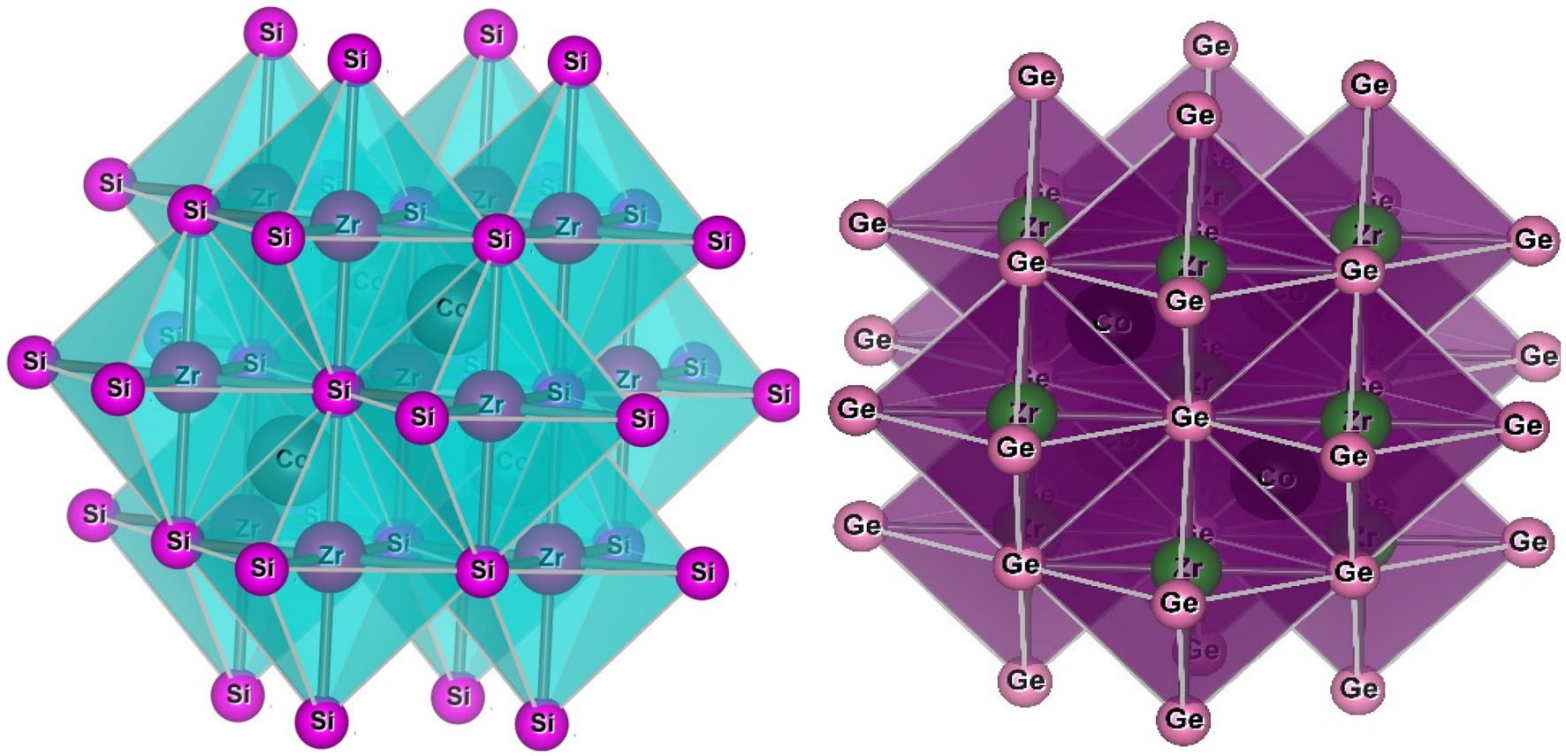
Pictorial representation of half Heusler CoZrSi and CoZrGe.

**Figure 2:**
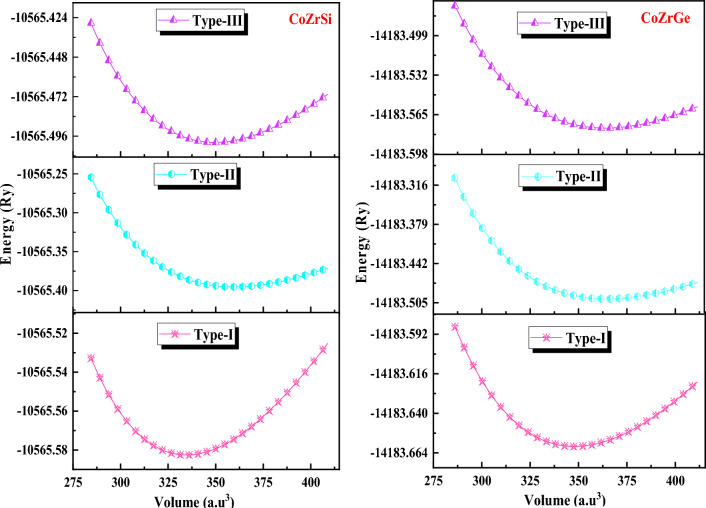
Energy as a function of volume in the 3 different phases for CoZrSi and CoZrGe.

**Figure 3:**
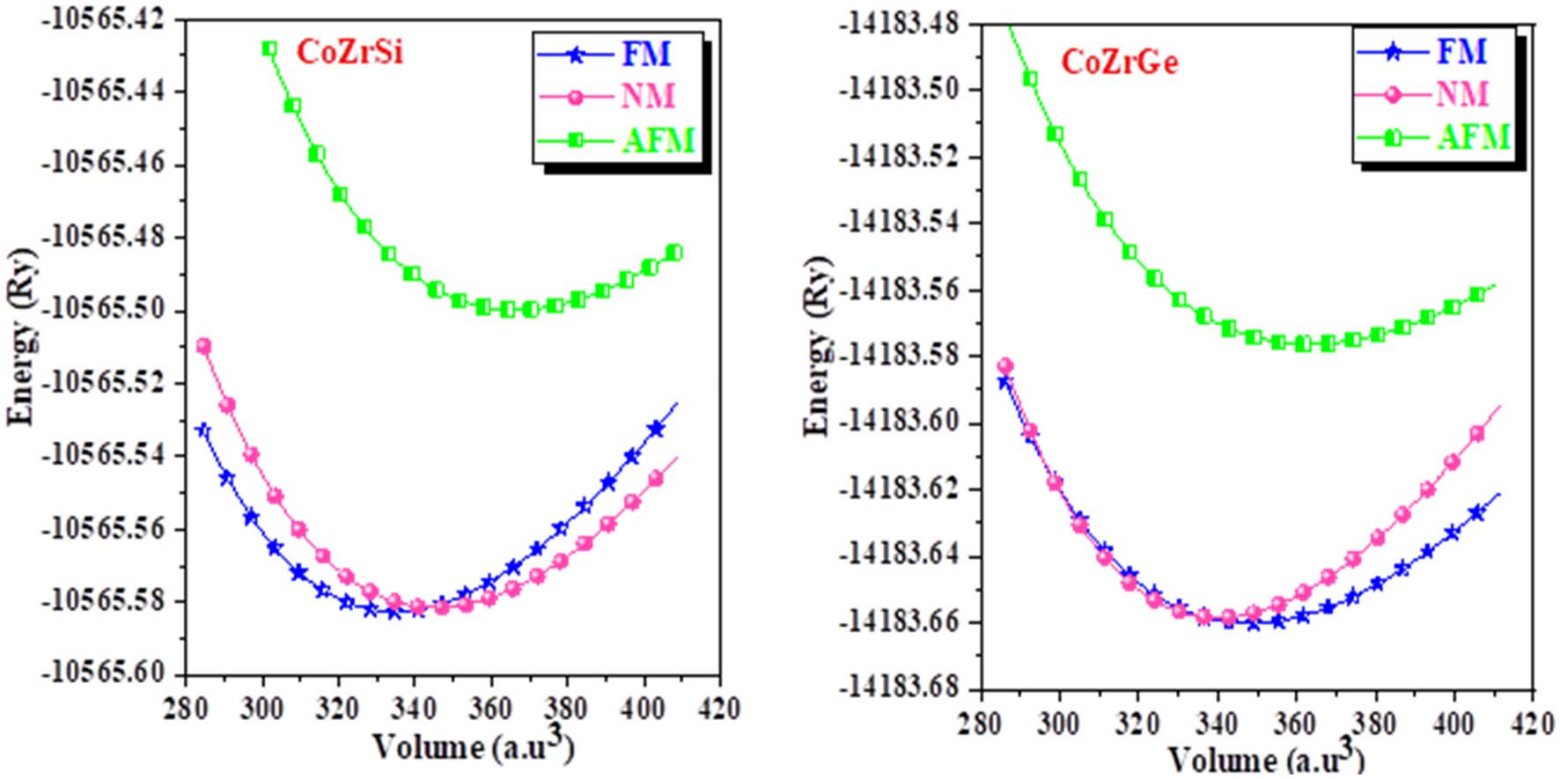
Energy as a function of volume in the FM, NM, and AFM phases for CoZrSi and CoZrGe.

The half-Heusler alloys exhibit cubic MgAgAs phase within the space group F-43 m. Within this space group, two configurations can exist: α-phase and β-phase. In the α-phase, the atom can occupy Wycoff positions 4a (0,0,0), 4b (0.5,0.5,0.5) and 4c (0.25,0.25,0.25), whereas, in the β-phase, the atom can be found at positions 4a (0,0,0), 4b (0.75,0.75,0.75) and 4c (0.25,0.25,0.25), we used in the calculation only the α-phase, all position of α-phase is recorded in Table 1. To determine the more stable geometry of these alloys, we examined their atomic locations in three distinct α-phase configurations: type I (4b,4a,4c), type II(4b,4c,4a) and type III (4c,4a,4b). Figure 1 illustrates the crystal structure with different atomic configurations. For each of the three configurations, we determined several structural characteristics, including the equilibrium lattice constant (a in Å), the bulk modulus (B in GPa), and its pressure derivative (B’_0_) at zero pressure as reported in Table 2. The geometric configuration of type I is found to be the most stable state for both of these alloys as delineated in Fig. 2 through three different types. The structure is optimized in three phases, i.e., spin-polarized (Ferromagnetic FM), non-spin-polarized (non-magnetic NM) and anti-spin-polarized (Antiferromagnetic AFM) as shown in Fig. 3. We calculated volume and total energy (E-V) results from the Birch-Murnaghan equation^39^ as laid out in Fig. 2.

**Table 1:** Wyckoff positions in various phases of half Heusler alloys.

α-phase	4a (0,0,0)	4b (0.5,0.5,0.5)	4c (0.25,0.25,0.25)
Type-I	Y	X	Z
Type-II	Y	Z	X
Type-III	Z	X	Y

**Table 2:** Calculated structural parameters lattice constant (a in Å), volume (V in a.u.^3^), bulk modulus (B in GPa), energy (E_0_ in Ry) of half Heuslers alloys CoZrSi and CoZrGe in three different types with FM, NM and AFM phases by using GGA approximation.

Alloys	Phase	a	V	B	B’_0_	E_0_
CoZrSi	Type-I	5.83	334.62	146.51	4.27	−10,565.59
	Type-II	6.10	381.31	86.42	5.03	−10,565.39
	Type-III	5.91	349.46	117.19	5.02	−10,565.46
	NM	5.82	333.42	146.23	4.23	−10,565.57
	AFM	5.93	345.86	125.23	4.96	−10,565.49
CoZrGe	Type-I	5.90	347.93	136.35	4.56	−14,183.66
	Type-II	6.18	398.64	84.57	4.88	−14,183.49
	Type-III	5.99	363.95	109.65	5.34	−14,183.56
	NM	5.91	347.33	138.03	4.29	−14,183.65
	AFM	5.89	367.45	114.35	5.13	−14,183.59
CoZrGe^33^	FM	5.91	348.56	136.69	4.64	−14,183.66
CoZrSb^34^	FM	6.31	369.45	148.63	4.39

### Electronic properties

Electronic characteristics determine the behaviour of the materials and bonds within the atoms. The electronic properties are examined in the optimized lattice parameter by calculating the material’s total energy, electronic band profile and density of the state (DOS) of the material. We also used different approximations like GGA and mBJ to estimate the behaviour of the material.

#### A: Band Profile

**Figure 4:**
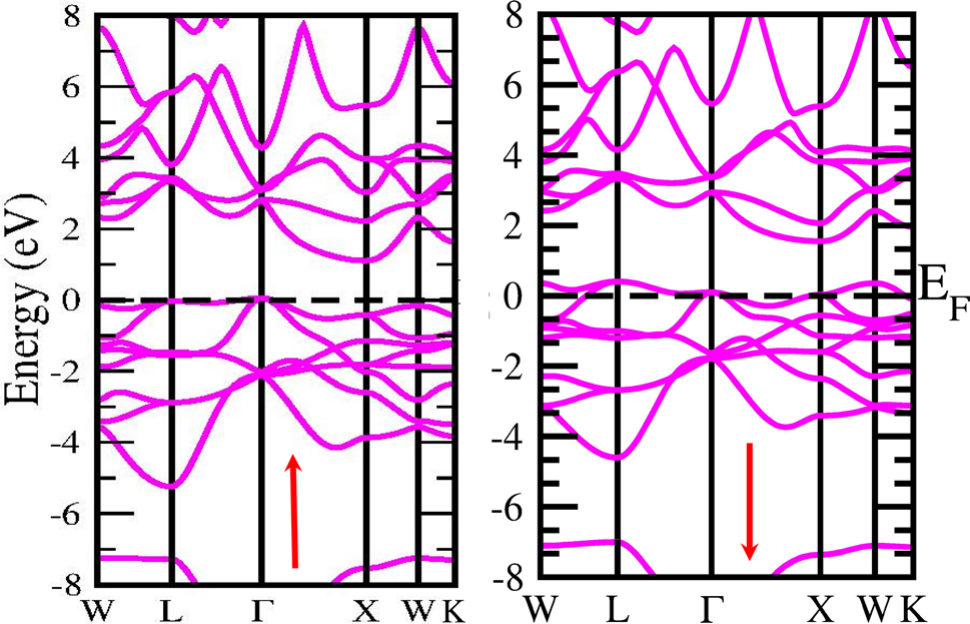
**S**pin polarised band structure of CoZrSi alloy by GGA approximation in spin up (↑) and spin down(↓) channel.

**Figure 5:**
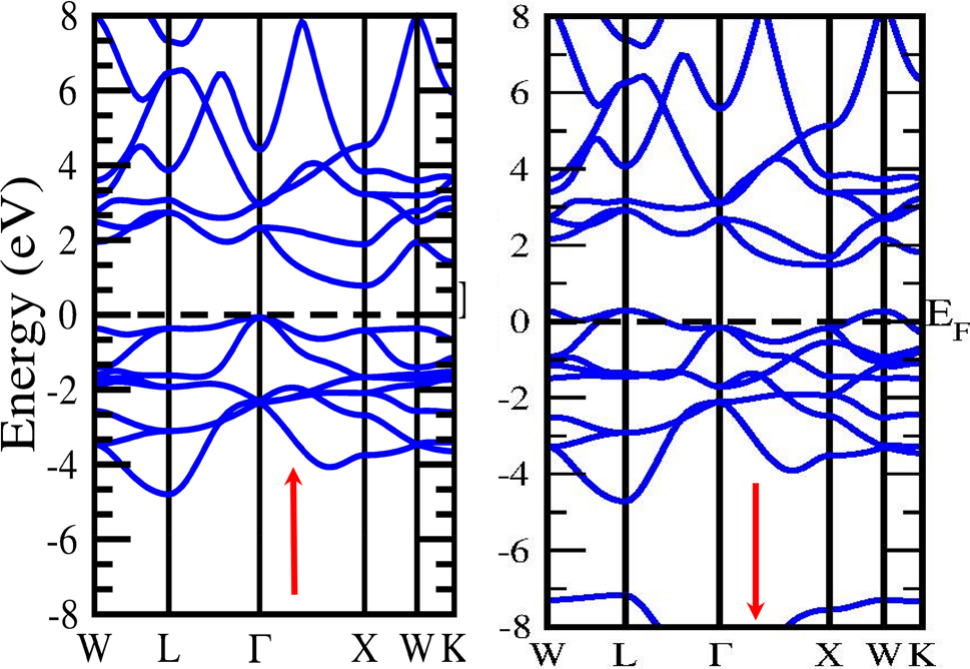
Spin polarised band structure of CoZrSi by mBJ approximation in spin up (↑) And spin down(↓) channel.

**Figure 6:**
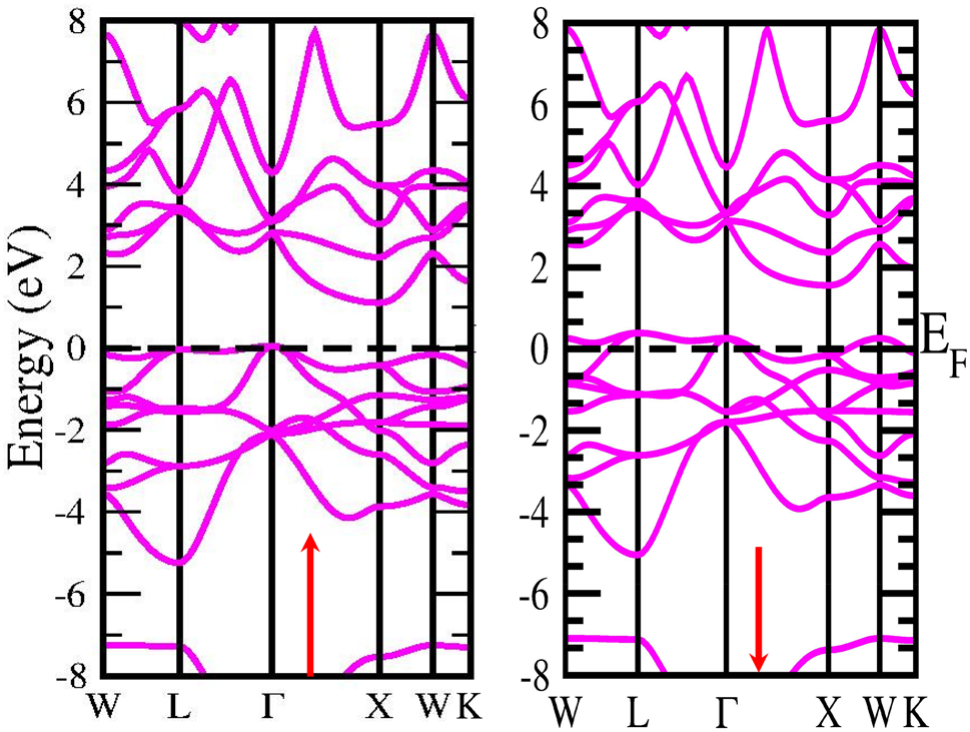
**S**pin polarised band structure of CoZrGe alloy by GGA approximation in spin up (↑) and spin down(↓) channel.

**Figure 7:**
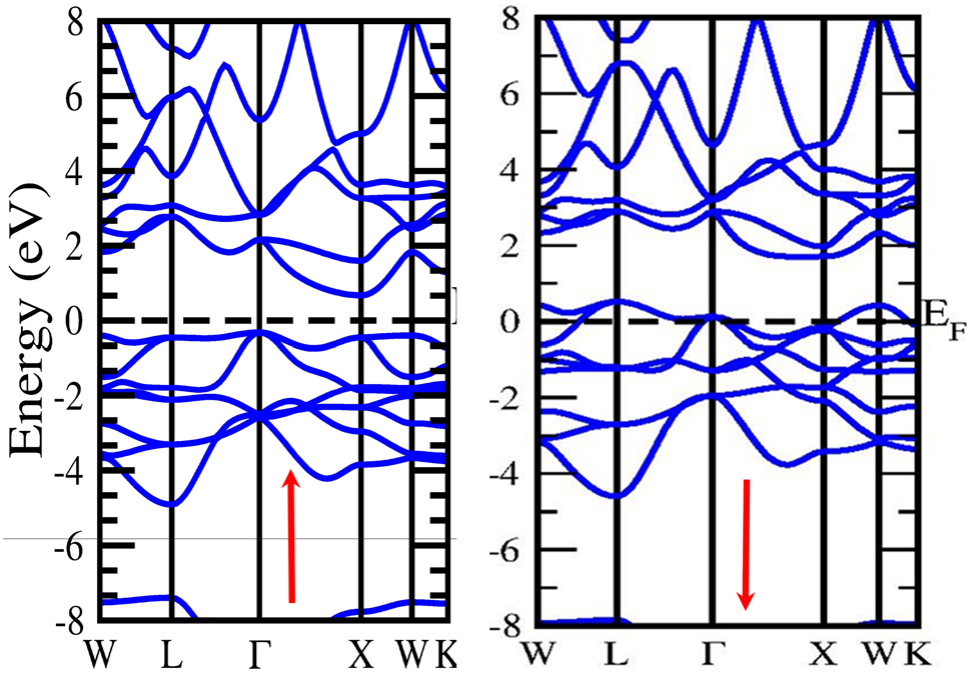
Spin polarised band structure of CoZrGe by mBJ approximation in spin up (↑) and spin down(↓) channels.

Analysing the electronic and magnetic properties of a material is a way to assess its suitability for technological applications. We have estimated the energy band Profile using the two schemes GGA and mBJ, in which mBJ gives the correct estimated energy band profile. The electronic band structure is crucial for identifying the nature of a material based on its electronic properties. It is used to define a system's valence and conduction bands as well as some other electrical phenomena. The band profile of half Heusler alloys CoZrSi and CoZrGe are laid out in Figs. 4, 5, 6 and 7 with GGA and mBJ approximation. These Figures show that the Co-based half Heuslers alloys cross the fermi level in up-spin and down-spin channels in GGA approximation which exhibits a metallic nature and in mBJ approximation energy shifted towards the negative energy from low energy regions, and hence it generates a gap. Thus, it shows a gap in the spin-up channel which indicates the semiconductor nature. Based on this outcome, it has been determined that these alloys exhibit a half-metallic character when analysed using the mBJ approximation method. We have introduced mBJ Because GGA underestimates the bandgap of the material. When we used the mBJ potential over GGA, it was found that both the alloys are half-metallic with an indirect bandgap of 1.05 eV and 0.92 eV for CoZrSi and CoZrGe at Г–X symmetric point in the mBJ scheme, as laid out in Figs. 3 and 4 and the calculated bandgap is reported in Table 6.

#### B: DOS (Density of states)

**Figure 8:**
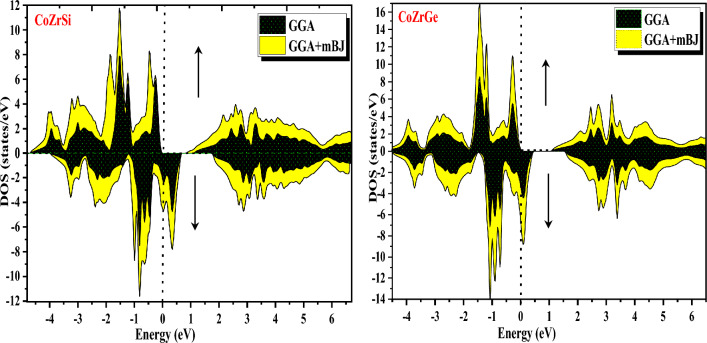
Calculated total density of state (DOS) in GGA and GGA + mBJ methods of CoZrSi and CoZrGe half Heusler alloys.

**Figure 9:**
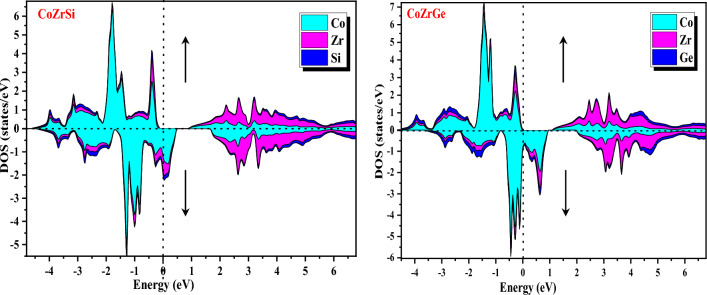
Calculated atomic density of state (DOS) in mBJ method of CoZrSi and CoZrGe half Heusler alloys.

**Figure 10:**
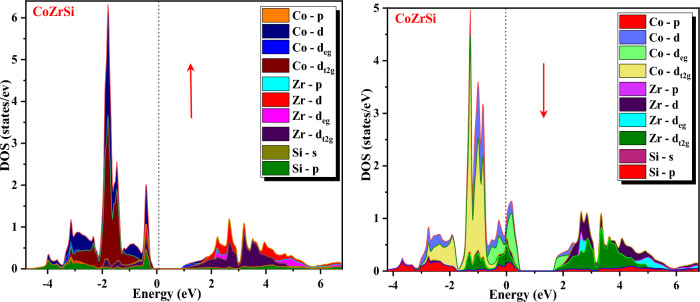
Calculated partial density of state (pDOS) in mBJ method for CoZrSi half Heusler alloy.

**Figure 11:**
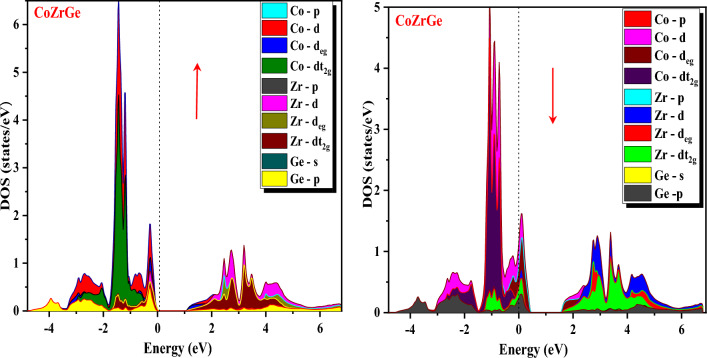
Calculated partial density of state (pDOS) in mBJ method for CoZrGe half Heusler alloy.

Furthermore, band structures of the alloys were also interpreted in terms of total DOS, partial DOS and atomic DOS as shown in Figs. 8, 9, 10 and 11. Figure 8 shows a comparison of the total DOS distribution for GGA and mBJ. The atomic density of states shows the distribution of individual atoms that can be plotted to further clarify the half-metallic character, which is shown in Fig. 9 for both the alloys for different atoms (Co, Zr, Si and Ge). The pDOS provides better results for understanding the nature of the bands of an alloy's metallic or semiconducting nature. The pDOS of CoZrSi and CoZrGe alloys under mBJ approximation are shown in Figs. 10 and 11. Based on the Fig. 8 analysis, the primary contributor to total state densities in the negative energy range is cobalt. Predominantly, cobalt and zirconium elements make significant contributions, as anticipated, owing to the electron carriers in transition metals being d-orbitals. This phenomenon occurs because the energy values of d-orbitals are closely aligned with the Fermi level, elucidating these contributions. Notably, the Silicon and Germanium element displays a sharp peak in the energy range of −12 to −10 eV, indicating a considerably low contribution of IV-A group elements around the Fermi level. Figure 9 delves into the examination of contributions from the elements' orbitals and atomic additives. The primary contributions from orbitals are highlighted by dashed lines. As expected, cobalt and zirconium, with their predominant d-orbitals, are the major contributors to electron densities. Conversely, Germanium, belonging to the IV-A group, predominantly employs s-orbitals or p-orbitals as its main electron carriers. Furthermore, the distinct peaks observed in Cobalt and Zirconium correspond to d-orbitals, while in the Germanium element, the sharp peak is attributed to the s-orbital. Significantly, this s-orbital, despite its low energy, appears in an energy region far from the fermi energy level. The results observed in Figs. 10 and 11 affirm the anticipated outcomes, validating the expected contributions from specific orbitals in Cobalt, Zirconium, Silicon and Germanium elements. The plots illustrate that the d-states of transition metals show metallic behaviour in the spin-down channel. However, in the spin-up channel, these d-states undergo splitting at the Fermi level, leading to the semiconducting nature of alloys. The d-d hybridization is profoundly responsible for splitting the d-state and forming the band gap. According to this, alloys are half-metallic, exhibiting 100% spin polarization.

#### C: Magnetic property

The magnetic moment of a compound plays a major role in studying the magnetic properties of it. The spin magnetic moment of half Heusler alloys has been explained by the Galanakis model^50,51^, according to the variance between up-spin and down-spin states. Table 3 depicts the computed total magnetic moment along with the individual magnetic moment of Co, Si, Ge, and Zr atoms. According to the Slater-Pauling Rule (SPR), the estimated values of the magnetic moment for half Heusler should be an integer value. The Slater-Pauling Rule (SPR) is interpreted as^52^;

**Table 3:** Total, interstitial and individual magnetic moment (μB) and band gap (eV) of CoZrSi and CoZrGe half Heusler alloys.

Alloys	Methods	Co	Zr	Si/Ge	Int	Total	Band Gap
CoZrSi	GGA	0.55	0.12	0.04	0.27	1.00	0.00
	mBJ	0.70	0.05	0.04	0.18	1.00	1.05
CoZrGe	GGA	0. 75	0. 04	0. 05	0.14	0.98	0.00
	mBJ	0.58	0.12	0.05	0.23	0.99	0.92

$${M}_{T}=({Z}_{t }- 18)$$

Here, Z_t_ is the total amount of outer shell electrons, and M_T_ stands for the total magnetic moment^53^. This technique involves deducting 24 from the total valence electrons for full Heusler alloys and 18 from the total valence electrons for half Heusler alloys. As for the electronic configurations of the elements Co, Zr, Si, and Ge, they are as follows:

$${\text{Co}}: \, [{\text{Ar}}\left] {{\text{ 3d}}^{{7}} {\text{4s}}^{{2}} ,{\text{Zr}}: \, [{\text{Kr}}} \right]{\text{ 4d}}^{{2}} {\text{5s}}^{{2}} ,{\text{Si}}: \, [{\text{Ne}}\left] {{\text{ 3s}}^{{2}} {\text{3p}}^{{2}} {\text{and Ge}}: \, [{\text{Ar}}} \right]{\text{ 3d}}^{{{1}0}} {\text{4s}}^{{2}} {\text{4p}}^{{2}} .$$

In the CoZrSi and CoZrGe alloys, the total count of valence electrons (Zt) is 17. According to the SP rule (M_T_ = Z_t_—18), the total magnetic moment for the CoZrSi and CoZrGe alloys is 1.00 μB. Both the GGA and mBJ approximations yield total magnetic moments of 1.00 μB, which aligns with our findings, consistent with the SP rule. Notably, the transition metal Co makes the most significant contribution to the overall magnetic moment.

#### D: Curie temperature

The band structure, DOS, and the numerical value of total magnetization support the alloys half-metallic nature. Another essential property of spin injectors from an application standpoint is T_C_. Hence, we have computed the T_C_ for the CoZrSi and CoZrGe HH alloys using the mean field approximation (MFA)^54^. The MFA states that the difference between the energies of NM and FM states is related to T_C_. The equation written below can be used to compute the T_C_.

$${T}_{C }= \frac{\Delta {E}_{NM-FM}}{3 {K}_{B}}$$

where, *K*_*B*_ is the Boltzmann constant and $\Delta {E}_{NM-FM}= {E}_{NM }- {E}_{FM}$ is the energy difference between the NM and FM phases. Table 6 provides a list of the estimated values of T_C_. The computed values of T_C_ are found to be greater than the ambient temperature, demonstrating the suitability of these materials for spintronic applications.

#### E: Cohesive energy

The factor cohesive energy (E_Coh_), formation energy and mechanical stability are used to determine a material's stability theoretically. The E_Coh_ of a material is defined as the energy needed to separate it into its constituent parts and indicates the binding strength of an alloy. It measures a material's stability^55^. Therefore, the cohesive energy is used to measure the intermolecular energy of substances. The formula below includes adding all the atomic energies and subtracting them from the alloy's total energy, which can be used to determine the E_Coh_ values of the alloys under consideration.

$${E}_{Coh }= \frac{\left({E}_{Co}+ {E}_{Zr}+{E}_{Si/Ge}\right)- {E}_{Tot}}{3}$$

where E_Tot_ is the total energy of the substance under consideration, while E_Co_, E_Zr_ and E_Si/Ge_ are the energies of single atoms. The stability of the structure will increase with the cohesive energy value. The computed values of E_Coh_ for CoZrSi and CoZrGe are recorded in Table 4.

**Table 4:** Computed value of Curie temperature T_C_ (K), the energy difference between FM and NM states $\Delta {E}_{NM-FM}$ (eV per formula unit), cohesive energy (eV per atom) and Formation energy (eV per atom) for Both HH alloys.

Alloys	$\Delta {E}_{NM-FM}$	T_C_	E_Coh_	ΔE
CoZrSi	0.21	810	16.02	−3.78
CoZrGe	0.14	579	19.41	−4.64

#### F. Formation energy

The enthalpy of formation energy (ΔE) of a compound is the energy change associated with the formation of one mole of the compound from its constituent elements in their standard states. It can be calculated using the following formula:

$$\Delta {\text{E }} = {\text{ E}}_{{{\text{Total}}}} - {\text{ aE}}_{{\text{A}}} {-}{\text{ bE}}_{{\text{B}}} {-}{\text{ dE}}_{{\text{x}}}$$

where, E_Total_ is the total energy of the compound, E_A_, E_B_, and E_X_ are the energies of the constituent elements, a, b, and d are the stoichiometric coefficients of the constituent elements. If the enthalpy of formation energy is negative, then the compound is stable. This is because the formation of the compound is associated with a release of energy. The enthalpy of formation energies of CoZrSi and CoZrGe were calculated using this formula and found to be −3.78 eV/atom and −4.64 eV/atom, respectively. These negative values indicate that CoHfSi and CoHfGe are both stable compounds.

CoZrSi and CoZrGe are stable compounds with negative enthalpy of formation energies. This means that they are likely to form spontaneously when their constituent elements are mixed together.

### Mechanical properties

Through the elastic constants, mechanical stability is revealed under different circumstances^40^. The Energy-strain relations are used to calculate the elastic constants by implementing modest amounts of strain to the equilibrium structure and examining how the total energy changes. This report attempts to estimate the elastic properties of CoZrSi and CoZrGe half Heusler alloys. As the materials possess cubic structures, it becomes necessary to compute the second-order derivative of Birch Murnaghan for elucidating other mechanical characteristics, thereby requiring C_11_, C_12_, and C_44_. The relevant stability criterion’s criteria can be expressed mathematically as C_11_ + 2C_12_ > 0; C_11_, C_12_ > 0; C_44_ > 0 and C_11_ > 0 are followed to define structural stability^41^. Both CoZrSi and CoZrGe alloys satisfy the criterion criteria for stability, proving that both cubic structure alloys are mechanically stable. All the elastic parameters are calculated in Table 5.

**Table 5:** Calculated elastic parameters for CoZrSi and CoZrGe alloys. Elastic constants (C_11_, C_12_, and C_44_ in GPa), Bulk modulus (B in GPa), Shear modulus (G in GPa), Young’s modulus (Y in GPa), Cauchy’s pressure (C_P_ in GPa), Anisotropy ratio (A), Pugh’s ratio (B/G) and Poisson’s ratio (σ).

Elastic parameters	CoZrSi	CoZrGe
C_11_	233.86	192.02
C_12_	100.96	108.77
C_44_	65.31	45.90
B = (C_11_ + 2C_12_)/3	145.28	136.52
G_V_ = (C_11_-C_12_ + 3C_44_)/5	65.76	44.08
G_R_ = 5C_44_(C_11_-C_12_)/{4C_44_ + 3(C_11_-C_12_)}	65.76	44.19
G = (G_V_ + G_R_)/2	65.76	44.13
Y = 9BG/(3B + G)	171.42	119.53
A = 4C_44_/(C_11_-C_12_)	0.98	1.10
σ = (3B-Y)/6B	0.31	0.35
C_P_ = C_12_–C_44_	35.67	62.87
B/G	2.21	3.09
G/B	0.45	0.32
ξ = C_11_ + 8C_12_/7C_11_ + 2C_12_	0.56	0.68
ρ (kg/m^3^)	5.96	7.17
V_t_ (10^3^ m/s)	3319.41	2480.43
V_l_ (10^3^ m/s)	6247.62	5218.54
V_m_ (10^3^ m/s)	3684.88	2771.96
Ɵ_D_ (K)	486.30	424.86
T_m_ $\pm$ 300 (K)	1935.39	1688.04

Another important factor, elastic anisotropy (A) plays a crucial role in determining the micro-cracks formation during the development process^42^. If A = 1, the crystal must be entirely isotropic; if A is less than unity then elastic anisotropy is predicted. As a result, it can be seen from the estimated values in Table 5 that both the CoZrSi and CoZrGe Heusler have a high anisotropy. Pugh's ratio (B/G) has a maximum value of 1.75 to categorise the plastic performance of a material^43^. B/G > 1.75 indicates ductility; otherwise, brittleness is indicated. These materials have a ductile nature because Pugh’s ratios for these alloys are higher than the critical value. Frantsevich's ratio (G/B), a second criterion for stability utilised by the materials^44^, whose equivalent to mathematical value is less than 1.06 indicates that these alloys have limited resistance to shear deformation.

Poisson's ratio (σ) is another component that is used to distinguish the alloys for brittle or ductile nature ^45^. The Poisson's ratio (σ) also indicates the nature of the bonding forces in the material. The alloy is still ductile if its value is more than 0.26; otherwise, it becomes brittle. The Poisson's ratio has a limit between 0.25 and 0.5 for the central force in solids. Our computed value shows that these alloys have central-type bonding forces. The brittle or ductile nature of the materials can be determined directly using Cauchy's pressure (C_P_)^46^. A positive value of C_p_ indicates a ductile nature, whereas a negative value indicates a brittle nature. Since the importance of (C_P_) for CoZrSi and CoZrGe are determined to be 35.67 and 62.83, respectively, it can be predicted that both alloys are ductile as shown in Table 5.

This Kleinman parameter (ξ) calculates internal strains for bond twisting relative to bond elongating and indicates the comparatively simple bond bending^47^. Reduced bond stretching and reduced bond bending in a structure show that ξ = 0 and ξ = 1, respectively. This parameter is 0.56 for CoZrSi and 0.68 for CoZrGe. Therefore, the mechanical properties that accompany elastic constant and their definition illustrate how resistant they are to a wide range of external forces, providing a suitable foundation for industrial applications.

The Debye temperature is calculated by using the average sound velocity $\nu$_m_

$${{\theta}_{D}= \frac{h}{k}{\left[\frac{3n}{4\pi }\left(\frac{\rho {N}_{A}}{M}\right)\right]}^{1/3}}\nu_{m}$$

Velocity $\nu$_m_ is defined through transverse velocity $\nu$_t_ and longitudinal velocity $\nu$_l_ and it is given by the following equation:

$${\nu }_{m}=\frac{1}{3}{\left(\frac{2}{{v}_{s}^{3}}+\frac{1}{{v}_{l}^{3}}\right)}^{- \frac{1}{3}}$$

We calculated the value of θ_D_ using the values of the essential parameters; it is equivalent to 486.30 K for CoZrSi and 424.86 K for CoZrGe. We found that it exhibits a declining tendency as Silicon and Germanium increase in size. Using Fine’s relation, we determined the melting temperature of these materials^48^ using an elastic constant (C_11_).

$${T}_{m }\left(K\right)=\left[553\left(K\right)+ \left(5.911\right){C}_{11 }\right]GPa\pm 300 K$$

For CoZrSi and CoZrGe, the calculated values of melting temperature (T_m_) are 1935.39 $\pm$ 300 K and 1688.04 $\pm$ 300 K, respectively. Since CoZrSi & CoZrGe have a high subscript value it is suggested that the material can maintain its ground state structure over a wide temperature range.

We have calculated the sound phase velocities for the longitudinal and transverse modes based on elastic constants. There are only three directions of elastic waves in cubic F-43 m symmetry, [111], [110], and [100]. As the waves travel in further directions, they are quasi-transverse or longitudinal waves^49^. Further, we have calculated the average velocity or Debye velocity (V_D_) by using the following relation:

$${\nu }_{D}=\frac{1}{3}{\left(\frac{1}{{v}_{l}^{3}}+\frac{1}{{v}_{t1 }^{3}}+\frac{1}{{v}_{t2 }^{3}}\right)}^{- \frac{1}{3}}$$

All the computed values of phase velocities are publicized in Table 6.

**Table 6:** Estimated sound velocities (m/s) along different directions.

Planes	Sound velocities	CoZrSi	CoZrGe
[100]	ν_l_ = √C_11_/ρ	6259.68	5173.53
	ν_t1_ = √C_44_/ρ	3308.13	2529.46
	ν_t2_ = √C_44_/ρ	3308.13	2529.46
	ν_D_	3673.71	2822.52
[110]	ν_l_ = √C_11_ + C_12_ + C_44_/2ρ	6244.66	5230.86
	ν_t1_ = √C_11_-C_12_/ρ	3336.40	2408.68
	ν_t2_ = √C_44_/ρ	3308.13	2529.46
	ν_D_	3687.58	2757.09
[111]	ν_l_ = √C_11_ + 2C_12_ + 4C_44_/3ρ	6239.64	5249.83
	ν_t1_ = √C_11_-C_12_ + C_44_/3ρ	3327.04	2449.60
	ν_t2_ = √C_11_-C_12_ + C_44_/3ρ	3327.04	2449.60
	ν_D_	3692.38	2740.01

### Thermodynamic properties

We estimated multiple thermodynamic potentials via the quasi-harmonic Debye model to describe the thermodynamic stability of the alloys. Hereby, Gibbs function is interpreted as^56^,

$$G^{* } \left( {V;P,T} \right) = E\left( V \right) + A_{Vib } \left[ {{\uptheta } \left( V \right);T} \right]$$

where E (V) denotes energy, A_Vib_ is the vibrational term, and Ɵ (V) is the Debye temperature. The specific heat capacity (C_V_), thermal expansion coefficient (α) and Grüneisen parameter (γ) are included in the predicted thermodynamic potentials within the range of pressure and temperature are 0–20 GPa and 0–900 K respectively.

**Figure 12:**
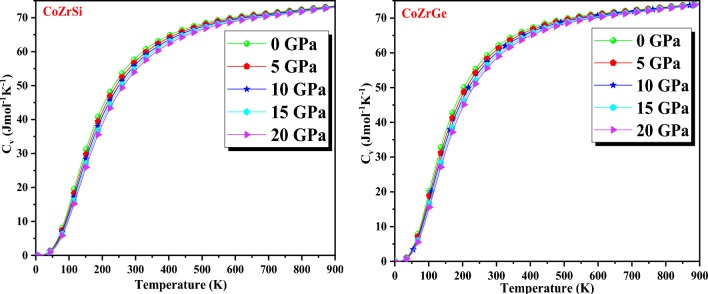
Variation of specific heat (C_v_) with temperature for CoZrSi and CoZrGe alloys.

Specific heat (C_V_) is a central parameter for assessing the intensity of phase transitions in lattice vibration that may take place in a material when temperature and pressure are applied. The Higher atomic or molecular mobility indicates the existence of high-temperature instability. The calculated value of C_V_ for CoZrSi and CoZrGe are shown in Fig. 12 which helps us to understand the impact of the increase in atomic vibrations caused by heat absorption. The graph shows that for different values of pressure, C_V_ tends to grow exponentially at low temperatures, whereas at high temperatures it attains a constant value. Every solid exhibiting the Dulong-petit law is defined by high-temperature variation^57^.

**Figure 13:**
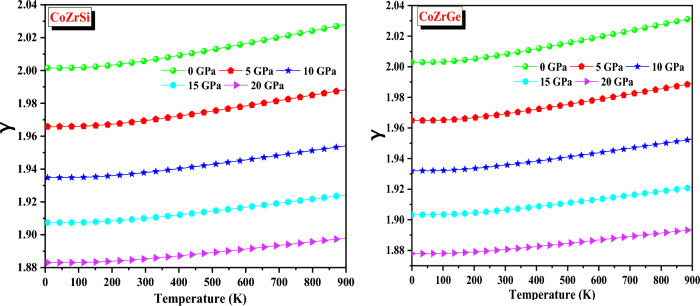
Variation of Grüneisen parameter (γ) with temperature for CoZrSi and CoZrGe alloys.

The Grüneisen parameter (γ) tells us about the phonon frequencies which are affected by crystal volume variation. It also reveals the amount of anharmonicity present in the source material. The fluctuation against temperature and pressure is shown in Fig. 13. At a lower temperature, these alloys exhibit a decreasing exponential pattern and at high temperature, they exhibit a constant value. The temperature effect outweighs the pressure effect, which exhibits nearly no change. The reported values of CoZrSi and CoZrGe at zero pressure (0 GPa) and 300 K are reported in Table 7.

**Table 7:** The evaluated value of thermodynamic parameters as Grüneisen parameter (γ), Specific heat (C_V_ in J mol^−1^ K^−1^), and Thermal expansion (α in 10^–5^ K^−1^) at zero pressure and room temperature.

Alloys	C_V_	γ	α
CoZrSi	58.29	2.006	1.36
CoZrGe	61.57	2.008	1.49

**Figure 14:**
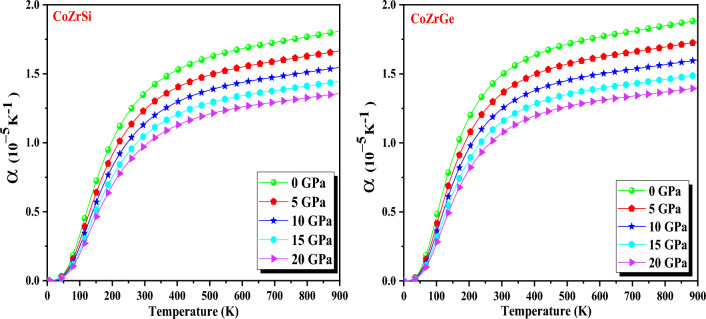
Variation of thermal expansion coefficient (α) with temperature for CoZrSi and CoZrGe alloys.

The thermodynamic equation of state is predicted by the way the thermal expansion coefficient (α) varies, making it significant from both a theoretical and an experimental approach. Figure 14 depicts the graphical difference for both alloys, with a fast-growing tendency at lower temperatures and a tendency toward constant values at higher temperatures. This is the result of the enharmonic effects at lower temperatures being suppressed. For all alloys, the modulus of the thermal expansion coefficient (α) shows a steady decline with pressure from 0 to 20 GPa. The (0 GPa) and 300 K values of α for CoZrSi and CoZrGe were computed as recorded in Table 7.

### Thermoelectric properties

Thermoelectric materials convert unused thermal energy into usable energy and can be used to create efficient and environmentally friendly energy sources^58^. This necessitates the material's ability to transmit charge and heat both effectively and optimally. Half Heuslers are one of the different kinds of alloys whose transport behaviour is being studied. The fluctuation of significant transport parameters is plotted from 50 to 900 K to demonstrate the material's thermoelectric performance. The various thermoelectric coefficients are distinguished as follows^59^:

$$\sigma = e^{2} \smallint {\Xi }\left( \varepsilon \right)\left( { - \frac{{\partial f_{0} }}{\partial \varepsilon }} \right)\partial \varepsilon$$

$$S = \frac{e}{T\sigma } \smallint {\Xi }\left( \varepsilon \right)\left( { - \frac{{\partial f_{0} }}{\partial \varepsilon }} \right)\left( {\varepsilon - {\upmu }} \right)\partial \varepsilon {\text{e}}$$

$$= \frac{1}{T} \smallint {\Xi }\left( \varepsilon \right)\left( { - \frac{{\partial f_{0} }}{\partial \varepsilon }} \right) \left( {\varepsilon - {\upmu }} \right)^{2 } \partial \varepsilon$$

**Figure 15:**
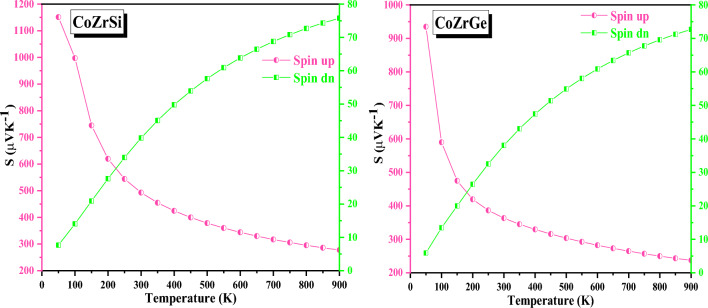
Variation in Seeback coefficient with temperature in both spin for CoZrSi and CoZrGe alloys.

The figure of merit formula ZT^60^ delivers a clear definition of the efficacy of the performance of the material. Electronic structure, particularly nearby to the fermi level, is the foundation for thermoelectric material’s efficiency. Compared to the p-states and d-states the Co and Zr atoms stay close to fermi energy. Consequently, electrons that are thermally excited from these states will undergo a transition to a state referred to as the Seebeck coefficient (S). Figure 15 illustrates how the Seebeck coefficient varies with temperature for both spin configurations. In both HH alloys, S is positive and suggests that both spin channels include p-type charge carriers (holes). The entire Seebeck coefficient was additionally estimated using two-current models, which is represented as^61^.

$$S = \frac{{\sigma \left( \uparrow \right)S\left( \uparrow \right) + \sigma \left( \downarrow \right){\text{S}}\left( \downarrow \right) }}{\sigma \left( \uparrow \right) + \sigma \left( \downarrow \right)}$$

**Figure 16:**
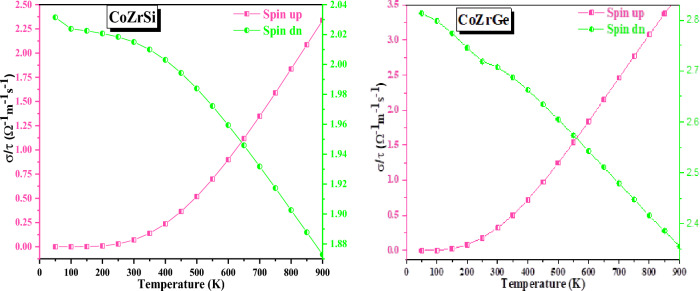
Variation in electrical conductivity with temperature in both spin for CoZrSi and CoZrGe alloys.

Figure 16 illustrates the relationship between electrical conductivity (σ/τ) and temperature (T). This graph demonstrates that as T grows and the concentration of carriers also rises, σ/τ is increased. Electrical conductivity based on carrier concentration is given by the equation σ = neµ^62^, where e and n denote electronic charge and mobility, respectively. Figure 16 reveals that increases in the spin-up channel and decreases in the spin-down channel, respectively, corroborate the semi-metallic behaviour. The value of Seeback coefficient at 300 and 900 K are recorded in Table 8.

**Table 8:** The computed value of electrical conductivity (σ/τ in 10^18^ Ω ^−1^ m ^−1^ s ^−1^), power factor (PF in 10^–5^ W/K^2^m), Seeback coefficient (S in µV K^-1^), electronic thermal conductivity (κ_Total_ in W/Km) and figure of merit (ZT) at 300 and 900 K for CoZrSi and CoZrGe alloy in spin up and down states.

Alloys	Spin states	S		σ/τ		κ_Total_		PF		ZT	
		300	900	300	900	300	900	300	900	300	900
CoZrSi	Up	492.92	277.41	0.07	2.33	6.58	6.38	8.48	49.71	0.10	0.51
	DOWN	39.82	75.76	2.01	1.87						
CoZrGe	UP	363.25	237.26	0.32	3.66	5.45	5.56	14.13	72.08	0.13	0.57
	DOWN	38.06	72.68	2.70	2.35						

Solids have thermal conductivity (κ_Total_) due to the two different components: (a) the movement of holes and electrons within the crystal that carries heat (κ_e_) and (b) the movement of phonons (κ_l_). These two components add up to the total thermal conductivity, which is given by κ_Total_ = κ_e_ + κ_l_. The carrier concentration is a significant factor that influences the total thermal conductivity in this case. The thermal conductivity increases the number of carriers. Slack's equation is utilised to estimate the lattice part of thermal conductivity since the BoltzTraP code assesses only the electronic component.

$${\upkappa }_{l} = \frac{{AM{\uptheta }_{D} V^{1/3} }}{{{\upgamma }^{2} n^{2/3} T}}$$

In this equation, A is constant with a value of 3.04 × 10^−8^, M is the average molar mass, ${\uptheta }_{D}$ is the Debye temperature, V is the average volume, ${\upgamma }$ is the Gruneisen parameter, n is the number of atoms in the primitive unit cell (where, n = 3 for half Heuslers and n = 4 for full/quaternary Heuslers) and T is the temperature. The calculated value of total thermal conductivity is recorded in Table 8 at 300 and 900 K for both the alloys.

The constant A is described as;

$$A\left( {\upgamma } \right) = \frac{{5.720 \times 0.847 \times 10^{7} }}{{2 \left[ {1 - \left( {\frac{0.514}{{\upgamma }}} \right) + \left( {\frac{0.228}{{{\upgamma }^{2} }}} \right)} \right]}}$$

**Figure 17:**
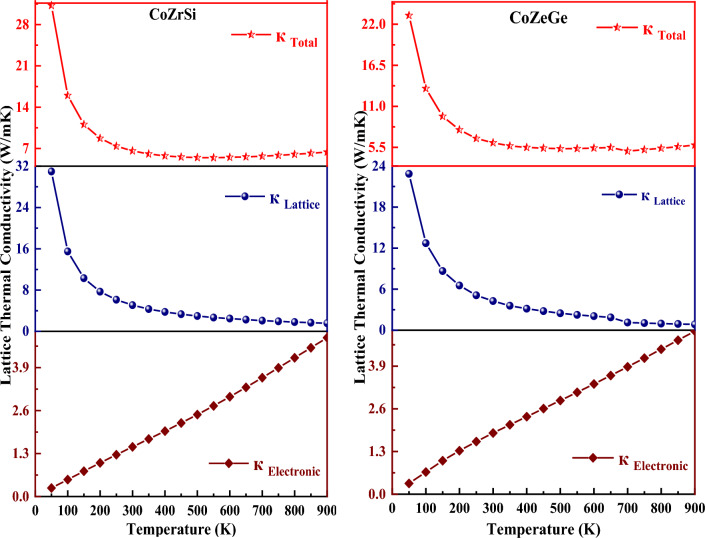
Variation in thermal conductivity (electronic, lattice, total thermal conductivity) with temperature for CoZrSi and CoZrGe alloys.

Near the Debye temperature, comparatively accurate results have been obtained. At low temperatures, it can anticipate a rough estimate of the necessary lattice conductivity. Figure 17 shows the fluctuations of thermal conductivity and total thermal conductivity, which reveals that the total lattice thermal conductivity of both forms was following a downward trend with a slight drop at low temperatures. Lattice thermal conductivity is prevalent at minimum temperatures which exhibits a fast decline with increasing temperature. The electrical component becomes increasingly prominent as the temperature rises, and this leads to an increase of κ_tot_. Lattice thermal conductivity generally decreases with the increase in temperature across the board for these materials. This phenomenon is caused by Umklapp processes, they show a 1/T dependence on the occupation of κ_l_ during phonon scattering at elevated temperatures^63^. The electrical thermal conductivity, on the other hand, increases as the temperature rises. Consequently, these half Heuslers carry high to low thermal conductivity for CoZrSi and CoZrGe as the temperature changes. This is because of the high anharmonicity they exhibit.

**Figure 18:**
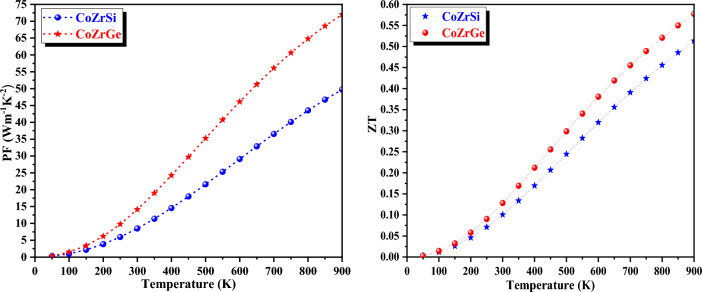
Variation in Power factor and figure of merits with temperature in both spin for CoZrSi and CoZrGe alloys.

An important thermoelectric factor is the power factor (PF), which is used to determine the amount of electrical energy produced. A Material is a strong candidate if it has a high-power factor. Figure 18 shows that over the selected temperature range, the PF of each of these alloys exhibits a rising trend. CoZrGe and CoZrSi both exhibit a rising PF trend from lesser values to higher values. The increase in electrical conductivity is the main source of the PF value's growing nature because the PF and electrical conductivity are related (PF = S2σ). These alloys have a high-power factor, indicating that they are composed for thermoelectric applications. The variation of power factor (PF) at 300 and 900 K is reported in Table 8 for both the alloys. The thermoelectric performance of a material is typically assessed using a metric called the figure of merit, denoted as ZT. The figure of merit (ZT) mathematically expressed as: $\it \it ZT = \frac{{S^{2} {\sigma T}}}{{_{{{\text{k}}}} }}$.

Figure 18 presents a graphical depiction of the figure of merit (ZT) as a function of temperature. At a temperature of 900 K, the observed ZT values for CoZrSi and CoZrGe alloys are 0.51 and 0.57, respectively. These substantial ZT values signify that both materials exhibit great potential for thermoelectric applications.

## Conclusion

We have summarized a comprehensive report on the Half Heuslers alloys CoZrSi and CoZrGe by illustrating their numerous fundamental properties. According to the DFT calculations, this crystalline structure is extremely stable because of its total ground state energy and cohesive energy. The band structures, which show spin-polarised properties reveal a half-metallic behaviour which is additionally confirmed by the integral value of the magnetic moment of these alloys. Elastic properties, including Cauchy pressure (C_P_) and Pugh’s ratio (B/G), reveal the ductile behaviour of these alloys. Thermal properties reveal the stability of these alloys under different temperatures. Additionally, we evaluated the thermoelectric properties, which indicate the suitability of these alloys for thermoelectric applications, where they can convert waste thermal energy into effective electrical energy. Generally, these Heuslers can be useful for applications in solid-state electronic devices and renewable energy.

## Data Availability

The datasets generated and/or analysed during this research work would be available from corresponding author on reasonable request.
